# Persistent sub-microscopic parasitaemia after ACT treatment of African children

**DOI:** 10.1186/1475-2875-13-S1-O20

**Published:** 2014-09-22

**Authors:** Colin Sutherland

**Affiliations:** 1 London School of Hygiene & Tropical Medicine, London, UK; 2 Hospital for Tropical Diseases, London, UK

## 

The efficacy of ACT for treating malaria patients infected with *Plasmodium falciparum* may be threatened by parasites with reduced responsiveness to artemisinins. Among 298 ACT-treated children from Mbita, Kenya, sub-microscopic persistence of *P. falciparum* on day 3 post-treatment was associated with subsequent microscopically detected parasitaemia at days 28 or 42.

Parasite density post-treatment was measured by duplex probe qPCR at days 1, 2 and 3. DNA sequences of resistance-associated parasite loci *pfcrt*, *pfmdr1*, *pfubp1* and *pfap2mu* were determined in the Mbita cohort before treatment, on days 2 and 3 after initiation of treatment and on the day of treatment failure.

Among 298 ACT-treated children from Mbita, Kenya, sub-microscopic persistence of *P. falciparum* on day 3 post-treatment was associated with subsequent microscopically detected parasitaemia at days 28 or 42. Parasites surviving ACT on day 2 or day 3 post-treatment were significantly more likely than the baseline population to carry the wildtype haplotypes of *pfcrt* (CVMNK at codons 72-76; P < 0.001) and *pfmdr1* (NFD at codons 86, 184, 1246; *P* < 0.001). In contrast, variant alleles of the novel candidate resistance genes *pfap2mu* (S160N/T; *P* = 0.006) and *pfubp*-1 (E1528D; *P* < 0.001) were significantly more prevalent post-treatment. No genetic similarities were found to parasites with reduced sensitivity to short-course artemisinin monotherapy, recently described in Cambodia.

We conclude that, among treated children in western Kenya, certain multi-locus *P. falciparum* genotypes are more likely to survive ACT at sub-microscopic level, and contribute to onward transmission and subsequent patent recrudescence. These surviving parasites may have an important public health impact that has been overlooked. The possible role of both innate and acquired immune mechanisms in this phenomenon will be discussed.

